# Confocal endomicroscopy: an additional approach in the watch-and-wait strategy for advanced rectal tumors

**DOI:** 10.1055/a-2598-4188

**Published:** 2025-06-13

**Authors:** Ana Victória Martins Lima, Fernanda Carvalho Franco, Guilherme Cutait de Castro Cotti, Carlos Frederico Sparapan Marques, Evandro Sobroza de Mello, Fauze Maluf-Filho, Adriana Vaz Safatle-Ribeiro

**Affiliations:** 1 Endoscopy Fellowship, Instituto do Câncer do Estado de São Paulo, University of São Paulo, São Paulo, Brazil; 2 Colorectal Surgery Division, Instituto do Câncer do Estado de São Paulo, University of São Paulo, São Paulo, Brazil; 3 Department of Pathology, Instituto do Câncer do Estado de São Paulo, University of São Paulo, Sao Paulo, Brazil; 4 Endoscopy Division, Instituto do Câncer do Estado de São Paulo, University of São Paulo, São Paulo, Brazil

A 73-year-old patient underwent a screening colonoscopy, which revealed an infiltrative lesion in the distal rectum. Histology confirmed the diagnosis of moderately differentiated infiltrative adenocarcinoma ( Fig. 1 ).

**Fig. 1:**
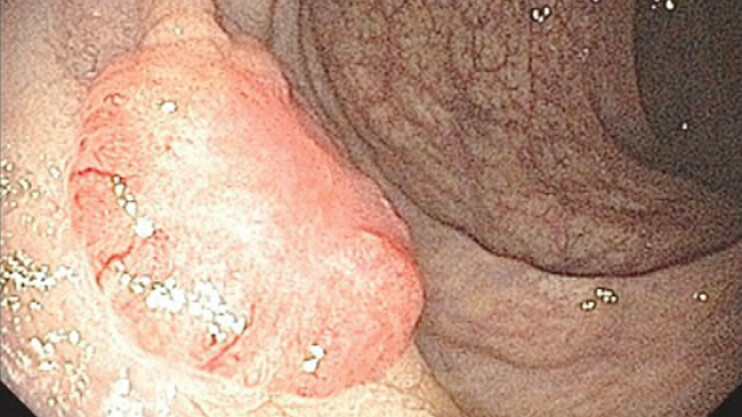
Infiltrative lesion in the distal rectum confirmed it as moderately differentiated infiltrative adenocarcinoma.

Locally advanced adenocarcinomas of the mid and distal rectum are best managed with neoadjuvant chemoradiotherapy, followed by surgery 1 . However, patients who achieve a complete clinical response may undergo a ‘watch-and-wait’ strategy for organ preservation, with strict follow-up to allow early detection in the event of tumor regrowth 2 3 .

The patient underwent neoadjuvant chemoradiotherapy, and 10 weeks after treatment, restaging exams were performed. Rectoscopy revealed a scar, and chromoscopy with narrowband imaging (NBI) demonstrated only increased vessels ( Fig. 2 ). Magnetic resonance imaging (MRI) revealed no residual lesion, suggestive of complete response.

**Fig. 2:**
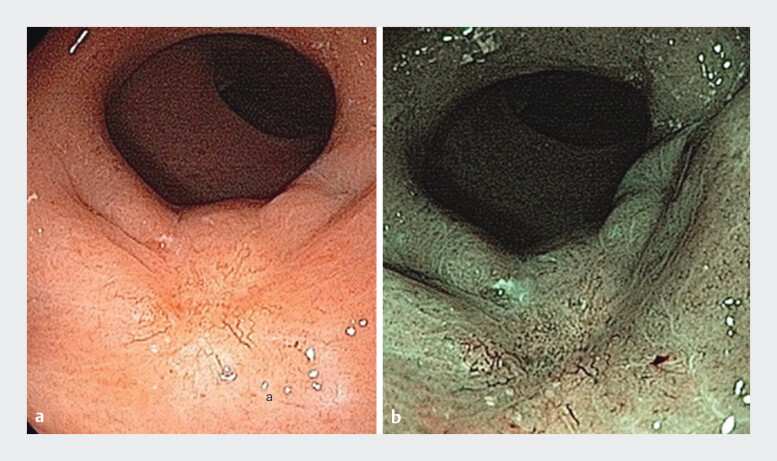
Scar identified on rectoscopy after neoadjuvant chemoradiotherapy treatment evaluated using **a** white-light imaging combined with **b** narrow-band imaging.

As part of the investigation, the patient underwent probe-based confocal laser endomicroscopy (pCLE), which is a real-time *, in vivo* method allowing a 1.000 times magnification, providing cellular and microvascular examination 4 , as demonstrated in Video 1 . It has been suggested that pCLE might be used during a watch-and-wait strategy for rectal neoplasia, avoiding immediate surgical treatment 5 .

Confocal endomicroscopy: a useful complementary method for diagnosing residual lesions following neoadjuvant chemoradiotherapy for advanced rectal adenocarcinoma.Video 1

pCLE revealed epithelial and vascular abnormalities indicative of a residual neoplastic lesion ( Fig. 3 ), and targeted biopsies confirmed the presence of adenocarcinoma. The patient underwent minimally invasive treatment by transanal endoscopic operation (TEO) ( Fig. 4 ), which provided a curative resection.

**Fig. 3:**
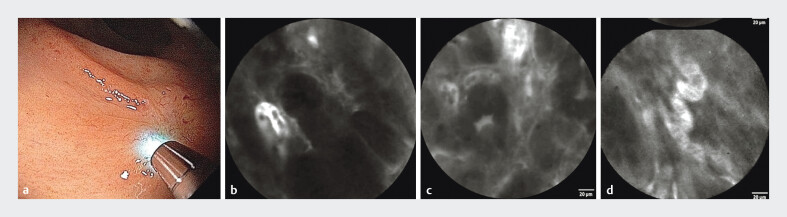
Epithelial and vascular abnormalities suggestive of a residual neoplastic lesion were identified using pCLE. **a** Probe positioned over the scar. **b** Irregular crypts along with thick and dark epithelium. **c** Back-to-back glands. **d** Dilated and tortuous vessels. Abbreviation: pCLE, probe-based confocal laser endomicroscopy.

**Fig. 4:**
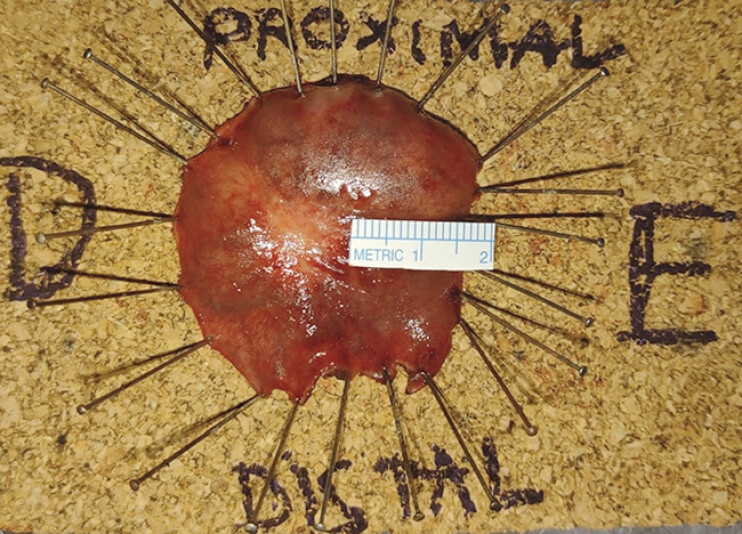
Specimen obtained through TEO confirmed a curative resection. Abbreviation: TEO, transanal endoscopic operation.

This case illustrates that pCLE could be a useful complementary method for diagnosing residual lesions following neoadjuvant chemoradiotherapy for advanced rectal adenocarcinoma.

Endoscopy_UCTN_Code_CCL_1AD_2AB

## Notes

Endoscopy E-Videos https://eref.thieme.de/e-videos
*E-Videos* is an open access online section of the journal *Endoscopy* , reporting on interesting cases and new techniques in gastroenterological endoscopy. All papers include a high-quality video and are published with a Creative Commons CC-BY license. Endoscopy E-Videos qualify for HINARI discounts and waivers and eligibility is automatically checked during the submission process. We grant 100% waivers to articles whose corresponding authors are based in Group A countries and 50% waivers to those who are based in Group B countries as classified by Research4Life (see: https://www.research4life.org/access/eligibility/ ). This section has its own submission website at https://mc.manuscriptcentral.com/e-videos .
